# Assessing the Diversity of *Beta vulgaris* L. ssp. *maritima* (Sea Beet) Populations in Egypt

**DOI:** 10.3390/plants13223152

**Published:** 2024-11-09

**Authors:** Asmaa A. Abdelhameed, Wafaa M. Amer, Najla A. Al Shaye, Mahmoud O. Hassan, Walaa A. Hassan

**Affiliations:** 1 Department of Botany and Microbiology, Faculty of Science, Beni-Suef University, Beni-Suef 62521, Egypt; asmaaadel.botany@science.bsu.edu.eg (A.A.A.); dr_mody1983_science@yahoo.co.uk (M.O.H.); azmeyw@gmail.com (W.A.H.); 2 Department of Botany and Microbiology, Faculty of Science, Cairo University, Giza 12613, Egypt; wamer@sci.cu.edu.eg; 3 Department of Biology, College of Science, Princess Nourah bint Abdulrahman University, P.O. Box 84428, Riyadh 11671, Saudi Arabia

**Keywords:** conservation, crop wild relatives, genus *Beta*, Mediterranean habitats, phenotypic variability, seed viability, taxonomy

## Abstract

Sea beet (*Beta vulgaris* L. subsp. *maritima* (L.) Arcang.) is a wild member of the Amaranthaceae family and a progenitor for all the cultivated beets (*Beta vulgaris* subsp. *vulgaris*). It is a source of stress-resistant genes, contributing 21 valuable traits to sugar beet through multiple breeding approaches. Despite its importance, the core morphological diversity of sea beet within the Egyptian Mediterranean coastal region has not yet been thoroughly explored. The field observations indicated notable morphological diversity among sea beet populations. This study investigated the morphological diversity of six sea beet populations along with their associated soil and climatic conditions in their primary habitats. Our morphometric investigations identified two varieties: *Beta vulgaris* subsp. *maritima* var. *glabra*, characterized by glabrous, erect, larger basal leaves, and *Beta vulgaris* subsp. *maritima* var. *pilosa*, distinguished by its hairy, prostrate form with smaller basal leaves. These varieties exhibited differences in their spatial distribution, showing high variations at the inter- and intra-population levels as well as the variety level. Soil parameters significantly influenced population morphological variability, which demonstrated a strong positive correlation with soil organic carbon. Our results highlight the need for precise survey and molecular characterization to secure these potential genetic resources from alteration and loss, especially in coastal habitats that are particularly sensitive to future climate change.

## 1. Introduction

*Beta vulgaris* L. is an annual or biennial herb with simple leaves and an erect-decumbent stem that can grow up to 100 cm long. Plants vary from glabrous to hirsute, and leafy branches from green to purplish-violaceous. The flowers are bisexual and green, typically arranged in clusters of (1-) 2–4 flowers forming glomerules and arranged along long interrupted spikes. Seeds measure 2–3 mm in diameter and are reddish-brown [1,2,3,4,5,6].

Crop breeding programs focus on wild relatives as a vital source of adaptive genetic diversity, which can enhance crop tolerance against biotic and abiotic stresses [7]. However, crop wild relatives (CWR) are currently threatened due to climate change and human overexploitation of plant species [8]. The genus *Beta* was identified in 2013 as one of the most important CWR genera in the global conservation priority list by Ascarini et al. [9]. Despite the importance of sea beets, there are few references to their anatomy and morphology as most research has focused on cultivated varieties [10]. Therefore, research is urgently needed to address this gap.

Meanwhile, the cultivated beets and wild sea beet populations are cross-compatible [11]. Such a gene flow from cultivated members to the wild taxon may affect the genetic structure of the nearest wild populations [12]. Therefore, conservation strategies for management and protection should focus on clearly defining these populations’ boundaries and recognizing the wild species’ genetic diversity to help distribution managers [13].

In heterogeneous habitats, soil conditions may affect plant morphology, especially nutrient contents [14,15]. Several studies have investigated the relationship between phenotypic variability and habitat heterogeneity [14,15,16,17,18]. According to Wieclaw et al. [19], habitat conditions should inform taxonomic studies, and the population ecology should consider the field conditions. Phenotypic variations within a given species, shaped by habitat conditions, may result in intra-specific differentiation and consequently the emergence of new taxa [16]. Despite the importance of sea beet populations as wild ancestors of the cultivated sugar beet and other cultivated beets, this taxon has not yet been thoroughly investigated.

Seeds are essential in conservation strategies since they initiate most restoration projects [20]. Additionally, several restoration programs rely on field-collected plants due to limited seed availability and low seedling survival rates [21]. However, this practice has been criticized for potentially harming donor populations and undermining restoration efforts [22]. Seed traits are valuable for species taxonomy and phytogeography owing to their conservation compared to other vegetative characteristics [23]. Trejo et al. [24] considered seed viability as a key indicator of the crop plant selection and inbreeding processes. According to Ulian et al. [25], it is important to understand the germination behavior of threatened plants for effective in situ and further ex situ conservation modeling.

*Beta vulgaris* ssp. *maritima*, commonly known as sea beet, is found throughout the Mediterranean region, along the coasts from Morocco to the southern part of the British Isles, in the Scandinavian region, and along the Atlantic coasts [26]. This plant is a rich source of magnesium, sodium, and vitamins A and C, and it is commonly used in herbal medicine for tumor treatments [27]. All parts of the sea beet—leaves, roots, and flowers—are edible [28]. Owing to their genetic variability, sea beet populations acquired disease resistance and adaptive traits that allowed them to thrive in challenging habitats [28,29]. These traits are useful for sugar beet crop breeding and adaptation [30]. Sea beet typically grows in clay soil or desertic habitats [7] and can adapt to soil with high salinity and water deficiency [31]. In Egyptian flora, the genus *Beta* L. is monospecific and is represented solely by the sea beet distributed along the Mediterranean coastal zone [2]. The Mediterranean habitats in Egypt and along the southern Mediterranean coast are increasingly affected by climate change and anthropogenic activities [32].

The field observations revealed morphological diversity among sea beet populations. We hypothesized that these morphological variations may be attributed to taxonomic identity, changes in microclimate, and/or soil properties. Thus, the main objective of this study was to decipher the variation patterns of inter and intra-specific populations of *Beta vulgaris* subsp. *maritima* and the environmental conditions supporting this variation.

## 2. Results

### 2.1. Taxonomic Identity for the Populations Studied

#### *Beta vulgaris* ssp. *maritima* (L.) Arcang. Comp. Fl. Ital. 593 (1882)

Syns. *Beta maritima* L., Sp. Pl., ed. 2, 322 (1762).

The morphological investigation of the studied sea beet populations based on thirty-five macro-morphological characters (Table 1) revealed that all these populations belong to *Beta vulgaris* ssp. *maritima.* This subspecies is distinguished from *Beta vulgaris* L. by its leaves: 2–12 × 1–5 cm, fleshy, glabrous, the basal in a rosette, ovate-cordate, long petiolate; cauline leaves: ovate-deltoid or rhombic, petiolate. Plants have inflorescence-dense spikes, sometimes long-branched, leafy, or leafless at the apex. Flowers are characterized by perianth segments 2–3 mm, green color, fleshy indurate in fruit, rounded triangular or spathulate, not or rarely incurved, without keel to ± strongly keeled, stigma ovate-lanceolate as long as the fruit diameter. Seeds are orbicular-reniform, 2–3 mm, smooth, red-dark brown. The studied populations of this subspecies were distinguished morphologically into two varieties. A key to distinguishing the *B. vulgaris* ssp. *maritima* varieties under Egyptian circumstances are:

- Plant glabrous, erect, with pale-green, basal leaves 2–12 × 1–5 cm, lower glomerule bracts exceeding by 10 times the glomerule length.................................. *Beta vulgaris* subsp. *maritima* var. *glabra*
- Plant hairy, prostrate, with dark green, basal leaves 2–7 × 1–3.5 cm, the lower glomerule bracts not exceeding by 10 times the glomerule length.................................................. *Beta vulgaris* subsp. *maritima* var. *pilosa*

### 2.2. Inter-Populations Plasticity

The descriptive statistics based on biometric measurements showed the most variable traits between the studied populations of *Beta vulgaris* ssp. *maritima*. Among these traits are the branch length (BL, which showed the highest coefficient of variation (CV = 61.86%), while the inflorescence length (InfL), number of branches/Stalk (NBS), number of inflorescences/Branch (NInfB), upper leaf petiole length (ULPL), and lower glomerule bract petiole length (LGBPL) showed CV > 50% (Table 1). The mean value of the plant length (PL) and the stalk length (SL) showed a high degree of data dispersion (phenotypic plasticity) with a high standard deviation of 27.24 and 20.26, respectively. On the other hand, low phenotypic plasticity was observed in lower glomerule length and width (LGL and LGW; respectively) with a coefficient of variation of 16.56% and 14.74%, respectively (Table 1).

### 2.3. Inter and Intra-Varieties Plasticity

The Kruskal–Wallis and ANOVA tests detected significant differences between the *Beta* populations studied in about 17 morphological characters (out of 35). The post hoc Mann–Whitney and the Tukey HSD test showed that the largest differences in characters between the following population pairs: var. *glabra* 2 vs. var. *pilosa* 1 and var. *glabra* 1 vs. var. *pilosa* 2, with significant differences in 13 and 12 morphological characters (out of the 35), respectively; var. *glabra* 1 vs. var. *pilosa* 1 and var. *glabra* 2 vs. var. *pilosa* 2 in 11 characters, and var. *glabra* 4 vs. var. *pilosa* 1 in 9 characters (Supplementary Tables S1 and S2).

**Figure 1:**
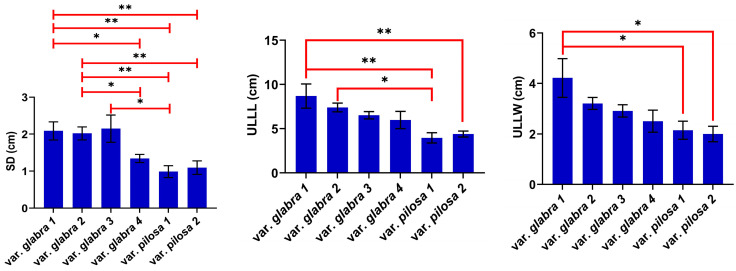

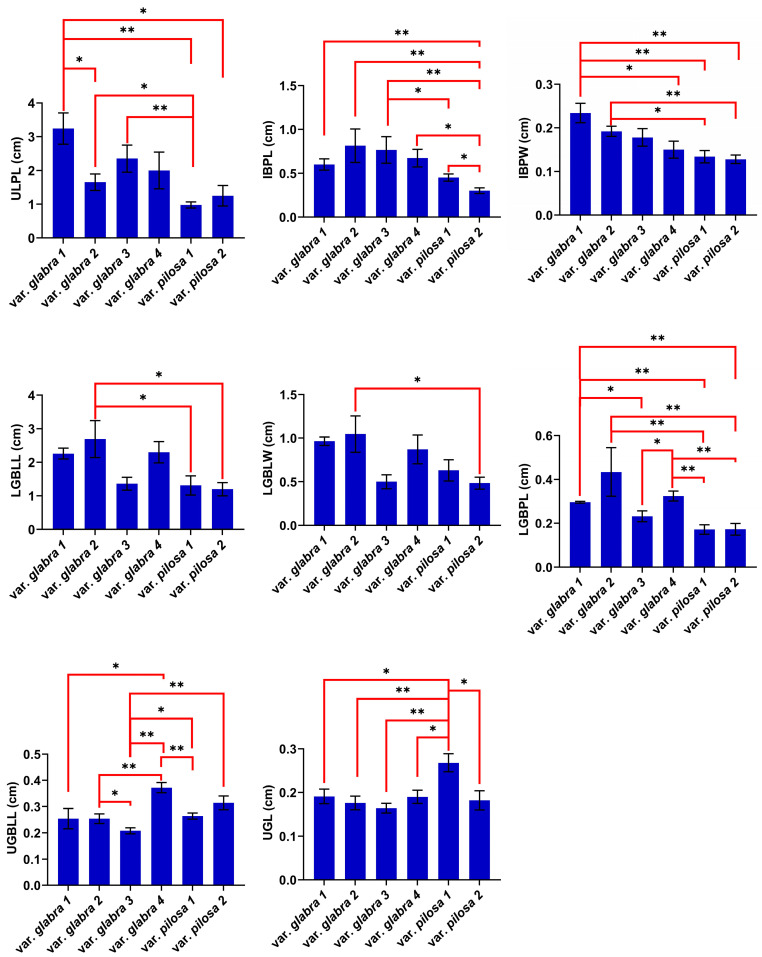
The most significant characteristics as mean values (±SEM) for the populations of the studied two sea beet varieties (*p*-value: * ≤ 0.05, ** ≤ 0.01).

The mean values of the most significantly different characteristics (based on Table 1) between the pairs of the studied varieties are outlined in Figure 1. The most significant differences were observed in the stalk diameter (SD) of var. *glabra* 1 and var. *glabra* 2 vs. var. *pilosa* 1 and var. *pilosa* 2. The individuals of var. *glabra* 1 compared with those of var. *pilosa* 1 and var. *pilosa* 2 also showed *p* value ≤ 0.01 in leaf characteristics including the upper leaf lamina and petiole lengths (ULLL and ULPL, respectively), inflorescence bract petiole width (IBPW), and lower glomerule bract petiole length (LGBPL). At the same time, the inflorescence bract petiole length (IBPL) varied between population pairs of var. *pilosa* 2 and *glabra* (1, 2 and 3). And the upper glomerule bract lamina length (UGBLL) showed variations between population pairs of var. *glabra* (2 vs. 4), var. *glabra* (3 vs. 4), var. *glabra* 3 vs. var. *pilosa* 2, and var. *glabra* 4 vs. var. *pilosa* 1 (Figure 1 and Supplementary Tables S1 and S2).

**Figure 2:**
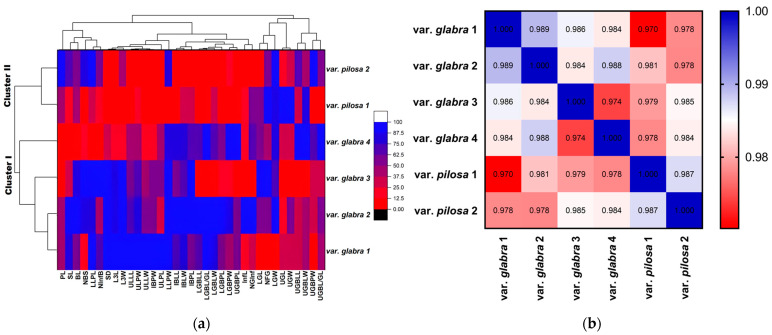
(**a**) Correlation-based cluster analysis using the unweighted pair group method for the studied *Beta* populations of the two varieties; (**b**) Spearman correlation illustrating the similarity values between the studied *Beta* populations of the two varieties.

The correlation-based cluster analysis (outlined in Figure 2a) separated the six-studied *Beta* populations into two clusters. Cluster I included populations of var. *glabra* (1, 2, 3, and 4). On the other hand, Cluster II included populations of var. *pilosa* (1 and 2). The highest similarity value was recorded between populations of var. *glabra* (1 and 2), var. *glabra* (2 and 4), and populations of var. *pilosa* (1 and 2): 0.989, 0.988, and 0.987, respectively. The lowest similarity value was 0.97, recorded between populations var. *glabra* 1 and var. *pilosa* 1 (Figure 2b).

**Figure 3:**
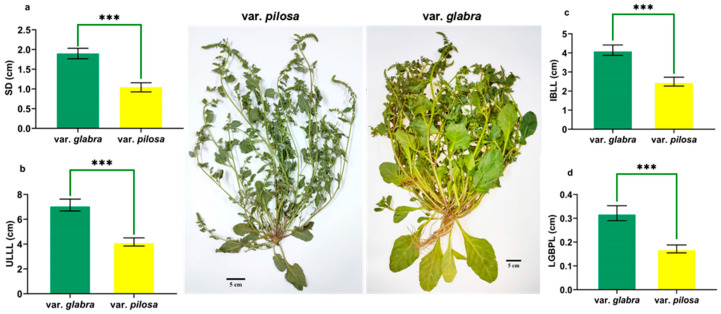
The two clusters representing the *Beta* populations studied for var. *glabra* & var. *pilosa* were distinguished based on cluster analysis using means (±SEM) of the most significant characters (**a**) Stalk diameter (**b**) Upper leaf lamina length (**c**) Inflorescence bract lamina length (**d**) Lower glomerule bract petiole length, *p*-value: *** ≤ 0.001.

Using the 35 morphological characteristics studied, the student’s *t*-test and the Mann–Whitney test revealed significant differences in 17 characteristics between the two clusters. (Supplementary Tables S3 and S4). Cluster I, representing var. *glabra*, is morphologically distinguished by thick stalk diameters (SD), longer upper leaf lamina length (ULLL), longer lower glomerule bract petiole length (LGBPL), and longer inflorescence bract lamina length (IBLL) compared to the populations in Cluster II representing var. *pilosa* (Figure 3, Supplementary Tables S3 and S4).

### 2.4. Soil Parameters Supporting the Beta Varieties

The results showed a significant variation in soil parameters among the six-studied populations (Table 2). The organic carbon and the sand percentages were significantly prominent for the var. *glabra* populations (1 and 2). Meanwhile, var. *glabra* populations (3) were correlated with high potassium, phosphorus, zinc, and soil water content. Var. *glabra* 4 populations prevailed in high electric conductivity, nitrogen, phosphorus, and potassium. Later, var. *pilosa* populations (1 and 2) were found to correlate with the highest percentages of silt and clay, respectively (Table 2).

### 2.5. Climatic Parameters Supporting the Beta Varieties

Table 3 outlined that relative humidity (%) and solar irradiance showed significant variation associated with var. *glabra* 1 and var. *pilosa* (1 and 2). Populations of var. *glabra* (2–4) only achieved the highest value of mean maximum temperature, while the increase in the rest of the data was for populations of var. *pilosa* (1 and 2).

### 2.6. Correlation Between the Morphological Characters and Soil Parameters

**Figure 4:**
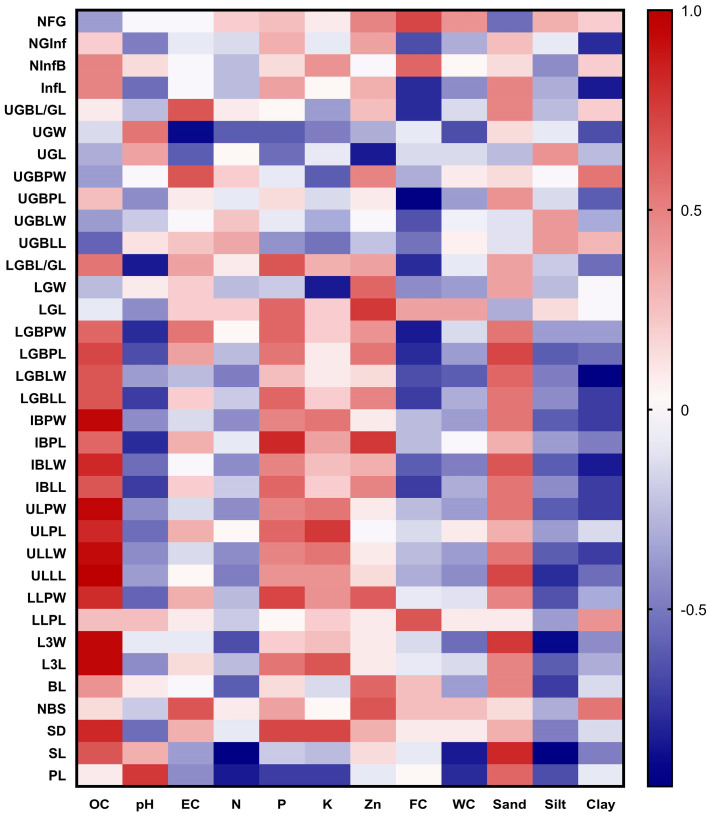
Spearman correlation heat map between the morphological characteristics of the *Beta* populations studied and the soil parameters. OC: organic carbon; pH potential hydrogen; EC: electrical conductivity; N: nitrogen; P: phosphorus; K: potassium; Zn: zinc; FC: field capacity; WC: water content.

The Spearman correlation heat map between the studied morphological characters with the soil parameters is outlined in Figure 4. This figure indicated a strong positive correlation between soil organic carbon and some morphological characteristics including stalk diameter (SD) and leaf dimensions (lamina length & width, petioles of lower and upper leaves, and inflorescence bracts). Inflorescence bract petiole length (IBPL) is positively correlated with phosphorus, although a negative correlation appeared between zinc and upper glomerule length (UGL). The upper glomerule bract petiole length (UGBPL) is negatively correlated with field capacity. The stalk length (SL) and lower leaf lamina length (L3L) are negatively correlated with the soil water content and the percentage of silt while being positively correlated with the percentage of sand.

### 2.7. Correlation Between the Morphological Characters and Climatic Parameters

**Figure 5:**
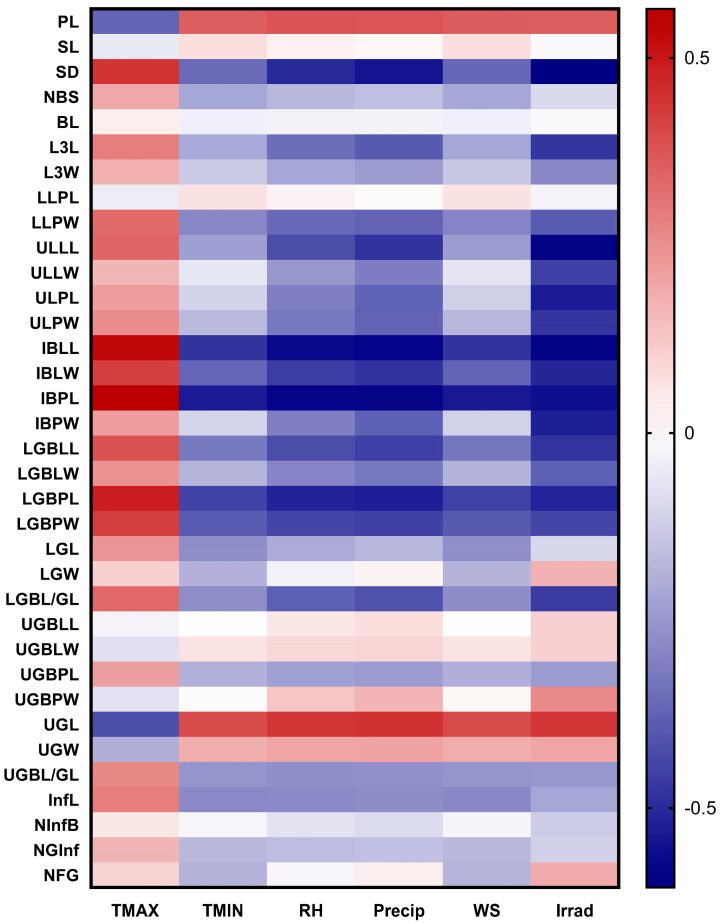
Pearson correlation heat map based on multiple linear regression analysis between the morphological characteristics of the *Beta* populations studied and climatic parameters. TMAX: maximum temperature; TMIN: minimum temperature; RH: relative humidity; Precip: precipitation; WS: wind speed; Irrad: solar irradiance.

The Pearson correlation heat map is based on regression analysis between the studied morphological characteristics with the climatic parameters (Figure 5). Leaf characteristics have a positive correlation with mean maximum temperature and a negative correlation with mean minimum temperature, relative humidity, precipitation, and wind speed. Among these characteristics are lower leaf petiole width (LLPW), inflorescence bract lamina length and width (IBLL and IBLW, respectively), lower glomerule bract lamina length (LGBLL), and the ratio of the lower glomerule bract length to the lower glomerule length (LGBL/GL). On the other hand, solar irradiance is negatively correlated with the dimensions of lower and upper leaves and inflorescence bracts and positively correlated with the plant and upper glomerule length (PL and UGL, respectively) (Figure 5).

### 2.8. Correlation Between the Beta Varieties with Soil and Climatic Parameters Studied

**Figure 6:**
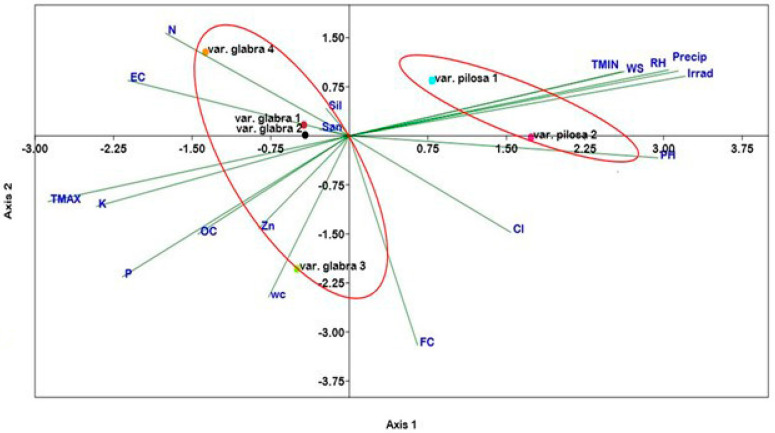
Canonical correspondence analysis tri-plot showing the possible relationship between the soil and climatic variables and the studied populations of the two *Beta* varieties. OC: organic carbon; pH potential hydrogen; EC: electrical conductivity; N: nitrogen; P: phosphorus; K: potassium; Zn: zinc; FC: field capacity; WC: water content; TMAX: maximum temperature; TMIN: minimum temperature; RH: relative humidity; Precip: precipitation; WS: wind speed; Irrad: solar irradiance.

Canonical Correspondence Analysis (CCA) (Figure 6) allows for the proper identification of the correlation between studied populations of *Beta* varieties and the measured soil and climatic parameters. The results of CCA indicated that axis 1 and axis 2 expressed about 71.13% of the total variance. The tri-plot showed that the two populations (1 and 2) of var. *pilosa* are positively correlated with minimum temperature, wind speed, relative humidity, precipitation, and solar irradiation and that the population of *pilosa* 2 is more correlated with pH (Figure 6). On the other hand, var. *glabra* (1, 2, and 4) is displaced in another group and positively correlated with N, EC, and the percentages of sand and silt. Finally, population var. *glabra* 3 is more correlated with soil field capacity, water content, Zn, P, and organic carbon.

### 2.9. Inter and Intra-Varieties Variability in Seed Germination

**Figure 7:**
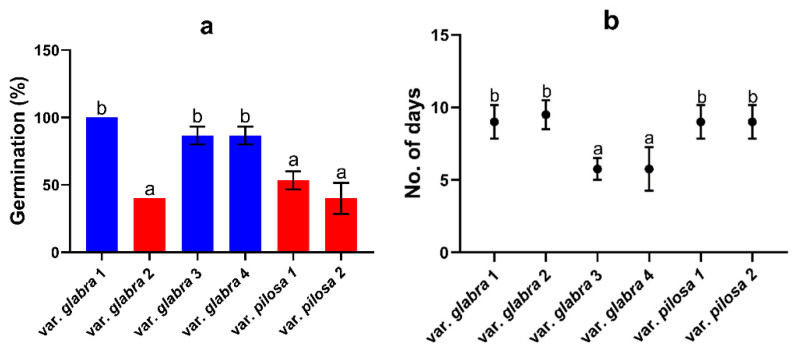
(**a**) Germination percentage (degree of freedom = 5, F value = 15.77, *p*-value = 0.000); (**b**) timing of germination (degree of freedom = 5, F value = 7.77, *p*-value = 0.000) for the studied *Beta* populations (±SEM); data with the same letter have no significant differences.

Our data showed that the percentage of seed germination varied significantly between populations (Figure 7a). The value was highest for the population of var. *glabra* 1 (100%) for about 10 days, while populations of var. *glabra* (3 and 4) remained for about 5 days to germinate 86.7% of the total seeds (Figure 7a,b). Populations of var. *pilosa* 2 and var. *glabra* 2 showed the lowest germination percentage of 40% for about 10 days after sowing. The morphological investigation of the juvenile individuals suggested maternal resemblance for the two studied varieties.

## 3. Discussion

Sea beet potentiality: *Beta vulgaris* L. subsp. *maritima* (L.) Arcang. is a wild ancestor of all cultivated beets including sugar beet. Among the importance of this wild ancestor is its ability to outcross and hybridize with the cultivated beet varieties [9,33]. Sea beet populations grow wild along the coastlines of several European nations [33]; in S. Europe it is threatened by genetic erosion, and accordingly it is recorded in the “European Red List of Vascular Plants” and listed as a “Vulnerable Red List” species in Portugal [34]. Despite this importance, the species diversity in Egyptian flora has not received adequate investigation.

Population identity: The taxonomic investigations of the studied sea beet populations based on thirty-five macro-morphological characteristics revealed that *Beta vulgaris* ssp. *maritima* (L.) Arcang. is distinguished into two varieties, namely *Beta vulgaris* ssp. *maritima* var. *glabra* and *Beta vulgaris* ssp. *maritima* var. *pilosa.* Both were recorded in Egypt and identified earlier as *Beta maritima* L. *var. glabra* Delile and *B. maritima* L. var. *pilosa* Delile [35].

This taxonomic treatment is supported by populations grouping into two clusters. Cluster I, representing var. *glabra*, is morphologically distinguished by thicker stalk diameter (SD), longer upper leaf lamina length (ULLL), longer lower glomerule bract petiole length (LGBPL), and longer inflorescence bract lamina length (IBLL) compared to the populations in Cluster II representing var. *pilosa* (Figure 3). The morphological analysis of the sea beet populations studied shows a great diversity, where the trait values decreased clearly from Cluster I (populations var. *glabra*) to the populations of Cluster II (populations var. *pilosa*). The plant habit also showed notable differences, from erect in var. *glabra* (Cluster I populations) to prostrate in var. *pilosa* (Cluster II populations). Cluster I populations (var. *glabra*) showed well-developed aerial parts in terms of number of branches, leaf length, and width, while the populations of Cluster II are prostrate with reduced leaf area and increased seed production strategies. Relevant results indicated variations in all the studied morphological traits including the transition from erect to prostrate habit for sea beet populations from Madeira Island; this variation clarified 93.3% of field variation using PCA [9].

Spatial distribution of the identified varieties: The current research showed that the identified varieties were different in their spatial distribution, whereby the populations of var. *pilosa* are in the southern position compared to the locations of var. *glabra* populations. However, the sea beet populations studied by Ascarini et al. [9] were not identified taxonomically, and they reported congruent results that populations from different geographical sites showed significant morphological variations. They related these variations to the environmental adaptations controlled by epigenetic factors. In converse, ARNAUD et al. [36] reported that there is no harmony between the spatial distribution and the genetic clustering of the sea beet population.

Morphological diversity and soil parameters: Wieclaw et al. [19], suggested that morphological and molecular investigations for taxonomic studies should be supported by habitat conditions. The Spearman correlation heat map indicated a strong positive correlation between soil organic carbon and some morphological characteristics including stalk diameter and leaf dimensions. Cluster I populations (var. *glabra*) showed well-developed aerial parts in terms of number of branches, leaf length, and width. These populations occur mainly within canal banks and borders of cultivated land, while the populations of Cluster 2 are prostrate with reduced leaf area and increased seed production strategies. Congruent data were reported by Burns [37], who related this morphological diversity to the alteration in coastal conditions and nutrient availability which may be induced by habitat variability. Sea beet possesses a high phenotypic and genotypic variability towards environmental conditions such as salinity and nutrient deficit [38]. Canonical Correspondence Analysis indicated that the sea beet populations of var. *glabra* were positively correlated with nitrogen, organic carbon, phosphorus, potassium, and zinc availability than var. *pilosa*. High nitrogen and phosphorus levels significantly increased leaf area and induced root size [39], whereas in poorer soil roots become smaller and more fibrous [40]. Potassium, phosphorus, and nitrogen are considered basic elements for plant growth and development [41,42]. Var. *pilosa* is positively correlated with minimum temperature, wind speed, relative humidity, precipitation, solar irradiation, and pH. Ascarini et al. [9] reported that the sea beet populations grow closer to the sea subject to wind, under saline-dry locations, and retain prostrate-habit individuals with smaller leaf areas and higher seed productivity. This is analogous to the studied var. *pilosa* populations. Sea beet, which has adapted to saline coastal habitats, favors moisture availability in the soil [43]. *Beta vulgaris* ssp. *maritima* typically prefers a slightly alkaline to neutral pH (around 6.0 to 7.5) for optimal growth [44].

Meteorological data: The meteorological data in this study reported warmer maximum temperatures and lower solar irradiance in populations of Cluster 1 than in Cluster 2. Grassein et al. [45] and Roux et al. [46] suggested that plants enhance their growth and light uptake under low solar irradiance and warmer conditions by producing larger leaves. This can be used to interpret the variation in leaf size between the two Clusters.

Germination in the studied varieties: In our study, the time of fruiting varied clearly between populations, and therefore the ripened glomerules were collected at different times. Var. *glabra* 3 and var. *glabra* 4 completed their fruiting in March with low minimum temperatures and precipitation. Therefore, they reported higher germination values and a low timing of germination, attributed to maternal season conditions during seed ripening. Wagmann et al. [47] reported that frost and/or short periods of drought during the winter, summer, and autumn seasons may release seed dormancy. This maternal condition variation will cause phenotypic variability in germination values between offspring [48].

## 4. Materials and Methods

### 4.1. Materials for Morphological Investigation

**Figure 8:**
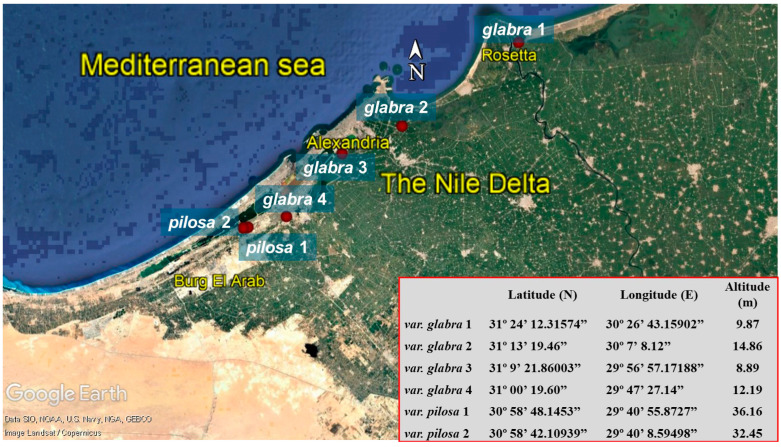
Map indicating the collection sites for the six studied sea beet populations.

Six populations of sea beet (*Beta vulgaris* L. subsp. *maritima* (L.) Arcang.) were collected along six localities representing the core morphological diversity of the studied taxa within the Egyptian Mediterranean coastal region (Figure 8). Five full flowering samples were randomly selected from each population during the winter and spring of 2020–2022. According to previous studies, thirty-five quantitative traits of the macro-morphological characteristics (Table 4) including stem, leaves, flowers, inflorescences, and fruits were used to address inter-population and inter-variety variability [9,10,49]. The identified *Beta* taxa were based on previous taxonomic treatments [1,2,3,4,5,6,50].

### 4.2. Soil Analysis and Climate Parameters

From each locality, three soil samples were randomly collected at 20 cm depth. Moreover, subsamples were collected for moisture content and field capacity determination [51]. In preparation for analysis, air-dried and sieved (2 mm sieve) soil samples were kept in dry and clean plastic bags. Soil water extract (1:2.5 *w*/*v*) was used for the estimation of soil pH and electric conductivity (EC) using a Professional Multi-Parameter Bench Meter) AD8000(. The soil particle size (texture) was determined using the international pipette method according to Piper [52]. According to Black [53], the rapid titration method was used to measure the soil’s organic carbon content. The available nutrients in the soil samples, including nitrogen, phosphorous, potassium, and zinc, were determined according to Cottenie et al. [54]. The meteorological data for the growing seasons of 2020–2022 were obtained from the Prediction of Worldwide Energy Resources project (POWER) [55]. Temperature (min. and max.), relative humidity (% RH), precipitation, wind speed, and solar irradiance were mainly considered.

### 4.3. Seed Viability Investigation

The seed viability and germination of the studied populations/varieties were investigated using full-ripened seeds. Seeds were collected from the studied populations/varieties during March 2022. The pot experiment was conducted in a protected area of Beni-Suef University’s experimental garden during the autumn and winter of 2022, using a completely randomized design with five replicates over three weeks. This experiment used air-dried surface soil from the El-Zaitoon area in Beni-Suef, located at coordinates 29°10′30.80″ N and 31°9′7.68″ E. The soil characteristics are as follows: texture: silty clay loam, pH: 7.76, electrical conductivity (EC): 329.75 μS cm^−1^, organic carbon content: 1.06%, and field capacity: 27.1%. For each population/variety, each pot was planted with forty seeds/pot. The pots were regularly irrigated as needed. The number of germinating seeds was scored daily during the germination period. After three weeks, the germinated seeds were scored, and one seedling/pot was kept for completing growth and further morphological analysis.

### 4.4. Data Analysis

Morphological traits were subjected to the Shapiro–Wilk test to verify the normality of data distribution. The parametric data were analyzed by one-way ANOVA followed by the Post-hoc (Tukey HSD test) to identify the different traits. Meanwhile, the Kruskal–Wallis and the Mann–Whitney tests were applied to the nonparametric data for multiple comparisons. Following the same steps, climatic and soil parameters were statistically analyzed. Specimens were sorted based on the complete morphological data set using correlation-based cluster analysis with the unweighted pair group method. Then, the average raw morphological data of the sorted specimens were applied to the student’s *t*-test and the Mann–Whitney test for comparison. To follow up on the relationship between environmental variables including soil and climatic factors and the growth habits of *Beta vulgaris* ssp. *maritima* populations/varieties, Spearman’s correlation test, multiple linear regression, and Canonical Correspondence Analysis (CCA) ordination were used. These analyses were performed by the IBM SPSS Statistics software version 25, GraphPad Prism version 8.0.1, the Past software v. 326b, and origin 2024b.

## 5. Conclusions

The study investigated the morphological diversity of six sea beet populations (*Beta vulgaris* subsp. *maritima*) in Egypt’s Mediterranean coastal region, focusing on potential taxonomic distinctions and their environmental correlates. *Beta vulgaris* ssp. *maritima* (L.) Arcang. were detected in Egypt in two varieties. *Beta vulgaris* subsp. *maritima* var. *glabra* Delile and *Beta vulgaris* subsp. *maritima* var. *pilosa* Delile are distinguished by trichomes, plant habit, and leaf size. Alongside the notable diversity in correlation with soil parameters, humidity, temperature, precipitation, wind speed, and solar irradiance, the seed germination percentage displayed considerable variations. These findings highlight the critical role of environmental conditions in shaping the phenotypic diversity of sea beet populations. Furthermore, the notable maternal resemblance of the offspring suggested a genetic variation between *Beta vulgaris* subsp. *maritima* var. *glabra* and *Beta vulgaris* subsp. *maritima* var. *pilosa*. The study underscores the need for continued research into sea beet conservation; therefore, we recommend conducting future molecular characterizations of *Beta* populations. This would provide a more comprehensive understanding of the adaptive strategies of this important crop wild relative and secure these potential genetic resources from the alteration and loss of the coastal habitats that are particularly vulnerable to the impacts of climate change. We also recommend conducting “common garden” experiments to assess phenotypic plasticity and the influence of soil characteristics on growth, as well as flower and seed production. Furthermore, comparing the leaf area index of different accessions will also help assess light capture and its relevance to the commercial production of sugar beet.

## Figures and Tables

**Table 1:** Morphological characters (cm) of the studied six *Beta vulgaris* subsp. *maritima* populations (*: significant at 0.05 level, **: the most significant at 0.01 level; Std.: standard, Min.: minimum, Max.: maximum, df: degree of freedom).

Trait	Mean	Std. Error	Std. Deviation	Median	Min.	Max.	% Coefficient of Variation	Interquartile Range	df	F-Value	*p*-Value
PL	58.32	4.97	27.24	57.75	16.5	115	46.72	39.2	5	1.65	0.18
SL	46.27	3.7	20.26	45.5	10.65	90.5	43.79	30.18	5	1.34	0.28
SD **	1.61	0.12	0.67	1.5	0.37	3.1	41.4	0.89	-	-	0.00
NBS	14.62	1.56	8.54	10.85	5.5	38	58.4	12.68	-	-	0.30
BL	14.17	1.6	8.77	15.58	1.2	34.7	61.86	14.89	-	-	0.49
L3L	9.66	0.64	3.52	8.98	4.5	18.2	36.43	5.22	-	-	0.06
L3W	4.3	0.3	1.66	4.2	1.8	8.8	38.49	2.25	-	-	0.21
LLPL	6.28	0.43	2.35	6.3	2.7	13.8	37.51	2.44	5	1.12	0.38
LLPW	0.29	0.01	0.08	0.3	0.14	0.45	26.86	0.11	-	-	0.15
ULLL **	6.16	0.42	2.31	5.5	2.38	11.6	37.48	2.93	5	5.19	0.00
ULLW **	2.83	0.21	1.16	2.78	1.1	5.9	40.96	1.13	5	3.59	0.01
ULPL **	1.91	0.2	1.07	1.5	0.26	4	56.04	1.37	-	-	0.01
ULPW *	0.21	0.01	0.05	0.2	0.1	0.3	21.99	0.07	-	-	0.05
IBLL *	3.6	0.24	1.33	3.57	1.4	6.1	36.94	2.19	-	-	0.02
IBLW	1.59	0.12	0.67	1.5	0.6	2.9	42.12	1.16	5	2.16	0.09
IBPL **	0.6	0.05	0.29	0.55	0.22	1.5	48.43	0.31	-	-	0.01
IBPW **	0.17	0.01	0.05	0.18	0.1	0.3	29.95	0.08	-	-	0.01
LGBLL **	1.85	0.16	0.87	1.7	0.7	4.2	46.87	0.93	5	0.01	0.01
LGBLW **	0.75	0.06	0.35	0.7	0.3	1.7	46.07	0.43	5	3.59	0.01
LGBPL **	0.27	0.03	0.14	0.27	0.1	0.84	50.69	0.11	-	-	0.00
LGBPW *	0.13	0.01	0.03	0.13	0.1	0.2	22.79	0.05	-	-	0.03
LGL	0.27	0.01	0.05	0.28	0.2	0.35	16.56	0.06	5	0.81	0.56
LGW	0.37	0.01	0.05	0.37	0.3	0.5	14.74	0.08	-	-	0.11
LGBL/GL *	7.87	0.67	3.67	7.22	1.85	17.2	46.63	5.77	-	-	0.02
UGBLL **	0.28	0.01	0.07	0.28	0.15	0.43	25.44	0.11	-	-	0.01
UGBLW	0.13	0.01	0.03	0.12	0.08	0.2	24.46	0.05	5	2.18	0.09
UGBPL	0.12	0.01	0.03	0.12	0.08	0.18	24.5	0.05	5	1.42	0.25
UGBPW *	0.07	0	0.01	0.07	0.05	0.1	20.86	0.02			0.04
UGL **	0.2	0.01	0.05	0.19	0.1	0.32	25.21	0.07	5	4.58	0.00
UGW	0.28	0.01	0.06	0.29	0.15	0.46	20.92	0.08	-	-	0.71
UGBL/GL	2.23	0.13	0.74	2.23	1.1	4.21	33.14	1.04	5	2.56	0.05
InfL	6.38	0.69	3.76	5.5	1.4	18.4	58.96	4.2	5	2.24	0.08
NInfB	6.12	0.65	3.57	5.5	1	15.6	58.34	4.4	5	1.34	0.28
NGInf	27.08	1.15	6.32	26.18	17	40	23.33	10.43	5	0.98	0.45
NFG *	2.37	0.21	1.16	3	1	4	48.98	2	-	-	0.02

**Table 2:** Soil analysis supporting populations of the studied varieties (Mean ± SEM); data with the same letters have no significant differences.

	Population	Var. *glabra* 1	Var. *glabra* 2	Var. *glabra* 3	Var. *glabra* 4	Var. *pilosa* 1	Var. *pilosa* 2	*p*-Value
Parameter								
Organic Carbon (%)		2.89 ^c^ ± 0.06	2.21 ^b^ ± 0.1	1.90 ^b^ ± 0.1	0.74 ^a^ ±0.07	0.42 ^a^ ± 0.01	0.72 ^a^ ± 0.04	0.000
pH		8.79 ^c^ ± 0.08	8.46 ^b^ ± 0.04	8.45 ^b^ ± 0.03	8.04 ^a^ ± 0.01	8.84 ^c^ ± 0.07	8.90 ^c^ ± 0.06	0.000
EC (µS cm^−1^)		947.25 ^b^ ± 10.9	975.75 ^b^ ± 14.01	2370 ^d^ ± 60.14	5785 ^e^ ± 58.67	557 ^a^ ± 12.06	1495.25 ^c^ ± 12.59	0.000
N (ppm)		32.65 ^b^ ± 0.26	21.89 ^a^ ± 0.06	43.62 ^e^ ± 0.36	109.85 ^f^ ± 0.25	41.67 ^d^ ± 0.39	38.35 ^c^ ± 0.30	0.000
P (ppm)		11.09 ^c^ ± 0.12	11.70 ^d^ ± 0.08	29.54 ^f^ ± 0.03	19.05 ^e^± 0.04	9.20 ^b^ ± 0.09	6.62 ^a^ ± 0.25	0.000
K (ppm)		933.12 ^d^ ± 3.17	437.48 ^b^ ± 3.31	1082.69 ^e^ ± 3.24	932.31 ^d^ ± 3.28	617.50 ^c^ ± 3.23	377.89 ^a^ ± 3.15	0.000
Zn (ppm)		1.01 ^a^ ± 0.04	4.46 ^e^ ± 0.01	3.45 ^d^ ± 0.04	2.60 ^c^ ± 0.00	1.43 ^b^ ± 0.03	2.43 ^c^ ± 0.05	0.000
Field capacity (FC%)		32.53 ^b^ ± 2.90	29.82 ^ab^ ± 2.21	47.49 ^c^ ± 1.36	22.93 ^a^ ± 2.01	33.75 ^b^ ± 1.75	32.70 ^b^ ± 2.13	0.000
Soil water content (WC%)		6.60 ^b^ ± 0.35	1.58 ^a^ ± 0.05	25.83 ^d^ ± 0.82	10.88 ^c^ ± 0.12	8.06 ^b^ ± 0.08	7.39 ^b^ ± 0.06	0.000
Sand (%)		84.35 ^b^ ± 1.98	91.89 ^b^ ± 3.02	51.39 ^a^ ± 8.27	54.72 ^a^ ± 4.01	47.49 ^a^ ± 10.35	67.43 ^ab^ ± 4.75	0.000
Silt (%)		4.74 ^a^ ± 1.46	1.91 ^a^ ± 0.40	28.91 ^ab^ ± 7.97	31.33 ^ab^ ± 3.71	39.86 ^b^ ± 13.18	11.12 ^ab^ ± 4.31	0.003
Clay (%)		10.91 ^a^ ± 0.52	6.20 ^a^ ± 3.23	19.70 ^bc^ ± 0.30	13.95 ^abc^ ± 0.33	12.66 ^ab^ ± 3.06	21.46 ^c^ ± 1.58	0.000

**Table 3:** Meteorological data supporting populations of the two studied varieties (Mean ± SEM); data with the same letters have no significant differences.

	Population	Var. *glabra* 1	Var. *glabra* 2	Var. *glabra* 3	Var. *glabra* 4	Var. *pilosa* 1	Var. *pilosa* 2	*p*-Value
Parameter								
Maximum temperature (°C)		28.49 ^a^ ± 0.52	31.49 ^b^ ± 0.43	31.49 ^b^ ± 0.43	31.49 ^b^ ± 0.43	27.31 ^a^ ± 0.34	27.31 ^a^ ± 0.34	0.00
Minimum temperature (°C)		15.51 ^b^ ± 0.1	12.82 ^a^ ± 0.14	12.82 ^a^ ± 0.14	12.82 ^a^ ± 0.14	15.66 ^b^ ± 0.07	15.66 ^b^ ± 0.07	0.00
Relative humidity (%)		68.42 ^b^ ± 0.35	65.72 ^a^ ± 0.56	65.72 ^a^ ± 0.56	65.72 ^a^ ± 0.56	70.73 ^c^ ± 0.45	70.73 ^c^ ± 0.45	0.00
Precipitation (mm.)		0.99 ^a^ ± 0.03	0.79 ^a^ ± 0.2	0.79 ^a^ ± 0.2	0.79 ^a^ ± 0.2	1.29 ^a^ ± 0.66	1.29 ^a^ ± 0.66	0.89
Wind speed (m/s)		4.12 ^b^ ± 0.04	3.22 ^a^ ± 0.01	3.22 ^a^ ± 0.01	3.22 ^a^ ± 0.01	4.19 ^b^ ± 0.01	4.19 ^b^ ± 0.01	0.00
Solar irradiance (MJ/m^2^/day)		20.43 ^a^ ± 0.03	20.43 ^a^ ± 0.03	20.37 ^a^ ± 0.02	20.37 ^a^ ± 0.02	21.46 ^b^ ± 0.05	21.46 ^b^ ± 0.05	0.00

**Table 4:** Abbreviations of the morphological characters (traits) used to study the *Beta vulgaris* L. subsp. *maritima* populations.

Trait	Abbreviation
Plant length	PL
Stalk length	SL
Stalk diameter	SD
Number of branches/Stalk	NBS
Branch length	BL
Lower leaf lamina length	L3L
Lower leaf lamina width	L3W
Lower leaf petiole length	LLPL
Lower leaf petiole width	LLPW
Upper leaf lamina length	ULLL
Upper leaf lamina width	ULLW
Upper leaf petiole length	ULPL
Upper leaf petiole width	ULPW
Inflorescence bract lamina length	IBLL
Inflorescence bract lamina width	IBLW
Inflorescence bract petiole length	IBPL
Inflorescence bract petiole width	IBPW
Lower glomerule bract lamina length	LGBLL
Lower glomerule bract lamina width	LGBLW
Lower glomerule bract petiole length	LGBPL
Lower glomerule bract petiole width	LGBPW
Lower glomerule length	LGL
Lower glomerule width	LGW
Lower glomerule bract length/Glomerule length	LGBL/GL
Upper glomerule bract lamina length	UGBLL
Upper glomerule bract lamina width	UGBLW
Upper glomerule bract petiole length	UGBPL
Upper glomerule bract petiole width	UGBPW
Upper glomerule length	UGL
Upper glomerule width	UGW
Upper glomerule bract length/Glomerule length	UGBL/GL
Inflorescence length	InfL
Number of inflorescence/Branch	NInfB
Number of glomerule/Inflorescence	NGInf
Number of flowers/Glomerule	NFG

## Data Availability

The original contributions presented in the study are included in the article/Supplementary Materials, further inquiries can be directed to the corresponding author.

## Supplementary Materials

The following supporting information can be downloaded at: https://www.mdpi.com/article/10.3390/plants13223152/s1, Table S1: Results of the Mann-Whitney test showing the significant differences (highlighted) in morphological traits between the pairs of the studied populations of the two studied varieties of *Beta* (1–4 = *glabra*, 5–6 = *pilosa*), the most significant traits according to Table 1 (*p*-value: ≤0.01) are red colored; Table S2: Results of the Tukey HSD test showing the significant differences (*p*-value: ≤0.05 highlighted) in morphological traits between the pairs of the studied populations of the two varieties of *Beta* (1–4 = *glabra*, 5–6 = *pilosa*), the most significant traits according to Table 1 (*p*-value: ≤0.01) are red colored; Table S3: Results of the Mann-Whitney test showing differences (*p*-value: ≤0.05 highlighted) between the two varieties of *Beta* resulting from hierarchical clustering using IBM SPSS Software, the most significant traits (*p*-value: ≤0.001) are red colored; Table S4: Results of the *t*-test showing differences (*p*-value: ≤0.05 highlighted) between the two varieties of *Beta* resulting from hierarchical clustering using IBM SPSS Software, the most significant traits (*p*-value: ≤0.01) are red colored.
