# Supplementary material for: Assessing the Diversity of Beta vulgaris L. ssp. maritima (Sea Beet) Populations in Egypt
Source: Plants (Basel). 2024 Nov 9;13(22):3152. doi: 10.3390/plants13223152 (PMC11597477; doi:10.3390/plants13223152)
Supplement: Supplementary file 1 [file plants-13-03152-s001.zip › plants-3270620-supplementary.pdf]

Table S1. Results of the Mann-Whitney test showing the significant differences (highlighted) in morphological traits between the pairs of the studied populations of the two studied varieties of *Beta vulgaris* subsp. *maritima* (1-4= *glabra,* 5-6= *pilosa)*, the most significant traits according to Table 1 (p-value: ≤ 0.01) are red colored.

Trait	Abbreviation	Population pairs														
		1x2	1x3	1x4	1x5	1x6	2x3	2x4	2x5	2x6	3x4	3x5	3x6	4x5	4x6	5x6
Stalk diameter	SD	0.830	0.599	**0.045**	**0.009**	**0.008**	0.750	**0.020**	**0.008**	**0.007**	0.093	**0.046**	0.071	0.113	0.289	0.592
Number of branches per stalk	NBS	0.346	0.602	0.117	0.295	0.175	0.917	0.075	0.173	0.753	0.251	0.175	0.754	0.917	0.347	0.076
Branch length	BL	0.347	0.917	0.347	0.754	0.917	0.602	**0.028**	0.347	0.675	0.117	0.917	0.917	0.602	0.117	0.917
Lower leaf lamina length	L3L	0.602	0.754	0.117	0.076	0.072	0.917	0.117	**0.009**	**0.008**	0.251	0.076	0.245	0.602	0.751	0.459
Lower leaf lamina width	L3W	0.602	0.917	0.295	0.347	0.396	0.602	0.117	**0.009**	**0.008**	0.465	0.251	0.666	0.602	0.245	0.090
Lower leaf petiole width	LLPW	0.590	0.915	0.832	**0.009**	0.914	0.915	0.750	**0.044**	0.590	0.748	0.160	0.598	**0.045**	0.916	**0.011**
Upper leaf petiole length	ULPL	**0.026**	0.169	0.072	**0.008**	**0.015**	0.172	0.754	**0.028**	0.530	0.465	**0.009**	0.059	0.175	0.402	0.175
Upper leaf petiole width	ULPW	0.164	0.070	**0.026**	**0.052**	**0.008**	0.671	0.517	0.131	**0.034**	0.525	0.347	0.075	0.598	0.395	1.000
Inflorescence bract lamina length	IBLL	0.465	0.754	1.000	0.076	**0.016**	0.209	0.602	**0.028**	**0.009**	0.402	0.251	0.116	**0.047**	**0.016**	0.600
Inflorescence bract petiole length	IBPL	0.449	0.525	0.396	0.090	**0.011**	0.917	0.754	0.059	**0.012**	1.000	**0.028**	**0.009**	0.175	**0.016**	**0.036**
Inflorescence bract petiole width	IBPW	0.203	0.138	**0.036**	**0.012**	**0.009**	0.746	0.090	**0.020**	**0.014**	0.289	0.113	0.091	0.525	0.523	0.827
Lower glomerule bract petiole length	LGBPL	0.911	**0.044**	0.410	**0.007**	**0.007**	0.094	1.000	**0.009**	**0.009**	**0.045**	0.094	0.142	**0.009**	**0.009**	1.000
Lower glomerule bract petiole width	LGBPW	0.831	0.286	0.140	**0.052**	0.219	0.205	0.399	**0.014**	0.066	**0.033**	0.219	0.408	**0.008**	0.105	0.700
Lower glomerule width	LGW	**0.020**	**0.011**	**0.008**	0.105	**0.052**	0.341	0.396	0.753	0.834	0.517	0.751	0.834	0.751	0.674	0.834
Lower glomerule bract length/ Glomerule length	LGBL/GL	0.754	0.116	0.465	0.117	0.117	**0.028**	0.917	**0.028**	**0.028**	**0.028**	0.753	0.463	**0.028**	**0.016**	0.834
Upper glomerule bract lamina length	UGBLL	0.598	0.599	**0.015**	0.599	0.463	**0.043**	**0.009**	0.750	0.115	**0.009**	**0.020**	**0.012**	**0.009**	0.094	0.114
Upper glomerule bract petiole width	UGBPW	**0.017**	0.238	**0.005**	0.519	**0.017**	0.913	0.214	0.163	0.086	0.445	0.338	0.055	0.113	0.110	0.080
Upper glomerule width	UGW	0.834	0.281	0.834	0.528	0.754	0.456	0.832	0.459	0.753	0.324	0.164	0.289	0.236	0.597	0.751
Number of flowers/Glomerule	NFG	**0.053**	**0.005**	**0.053**	**0.004**	**0.054**	0.212	0.577	0.343	0.827	**0.033**	0.513	0.125	**0.045**	0.827	0.178

Table S2. Results of the Tukey HSD test showing the significant differences (p-value: ≤ 0.05 highlighted) in morphological traits between the pairs of the studied populations of the two varieties of *Beta* (1-4= *glabra,* 5-6= *pilosa)*, the most significant traits according to Table 1 (p-value: ≤ 0.01) are red colored.

Trait	Abbreviation	Population pairs														
		1x2	1x3	1x4	1x5	1x6	2x3	2x4	2x5	2x6	3x4	3x5	3x6	4x5	4x6	5x6
Plant length	PL	1.000	0.933	0.829	1.000	0.738	0.928	0.820	1.000	0.749	1.000	0.947	0.226	0.854	0.139	0.706
Stalk length	SL	0.994	0.946	0.578	0.867	1.000	0.708	0.278	0.564	0.987	0.972	1.000	0.965	0.995	0.633	0.901
Lower leaf petiole length	LLPL	1.000	1.000	0.568	0.822	1.000	0.998	0.755	0.940	0.999	0.508	0.771	1.000	0.998	0.547	0.805
Upper leaf lamina length	ULLL	0.849	0.398	0.184	**0.003**	**0.009**	0.967	0.796	**0.050**	0.113	0.996	0.232	0.421	0.475	0.713	0.999
Upper leaf lamina width	ULLW	0.572	0.301	0.090	**0.026**	**0.015**	0.996	0.854	0.520	0.380	0.984	0.805	0.669	0.991	0.959	1.000
Inflorescence bract lamina width	IBLW	0.999	0.998	1.000	0.698	0.205	0.957	0.992	0.457	0.097	1.000	0.910	0.407	0.796	0.275	0.936
Lower glomerule bract lamina length	LGBLL	0.920	0.352	1.000	0.296	0.194	0.059	0.946	**0.046**	**0.026**	0.307	1.000	0.999	0.256	0.165	1.000
Lower glomerule bract lamina width	LGBLW	0.997	0.147	0.995	0.460	0.125	0.060	0.923	0.235	**0.049**	0.351	0.978	1.000	0.770	0.307	0.964
Lower glomerule length	LGL	0.830	0.552	0.914	0.861	1.000	0.996	1.000	1.000	0.925	0.981	0.993	0.699	1.000	0.972	0.944
Upper glomerule bract lamina width	UGBLW	1.000	0.686	0.583	0.942	1.000	0.618	0.652	0.966	1.000	**0.048**	0.205	0.686	0.976	0.583	0.942
Upper glomerule bract petiole length	UGBPL	0.999	0.856	0.879	0.985	0.949	0.681	0.971	0.918	0.831	0.256	0.996	1.000	0.514	0.387	1.000
Upper glomerule length	UGL	0.989	0.875	1.000	**0.044**	0.999	0.996	0.992	**0.011**	1.000	0.891	**0.003**	0.975	**0.041**	0.999	**0.019**
Upper glomerule bract length/Glomerule Length	UGBL/GL	1.000	0.998	0.445	0.635	0.991	0.983	0.591	0.488	0.999	0.233	0.869	0.907	**0.024**	0.791	0.303
Inflorescence length	InfL	0.177	1.000	1.000	1.000	0.998	0.116	0.281	0.173	0.081	0.996	1.000	1.000	1.000	0.983	0.999
Number of inflorescence/Branch	NInfB	0.992	0.974	0.663	0.825	1.000	0.778	0.931	0.986	1.000	0.253	0.394	0.895	1.000	0.834	0.941
Number of glomerules/Inflorescence	NGInf	0.798	0.998	0.973	0.995	0.995	0.553	0.995	0.973	0.497	0.846	0.934	1.000	1.000	0.801	0.905

Table S3. Results of the Mann-Whitney test showing differences (p-value: ≤ 0.05 highlighted) between the two varieties of *Beta* resulting from hierarchical clustering using IBM SPSS Software, the most significant traits (p-value: ≤ 0.001) are red colored.

Trait	Stalk diameter	Number of branches/Stalk	Branch length	Lower leaf petiole length	Upper leaf lamina width	Inflorescence bract petiole width	Lower glomerule bract petiole length	Lower glomerule bract petiole width	Upper glomerule bract petiole width	Inflorescence length
	SD	NBS	BL	LLPL	ULLW	IBPW	LGBPL	LGBPW	UGBPW	InfL
Mann-Whitney U	19.500	92.500	98.500	87.000	40.000	31.500	10.000	36.500	86.500	67.000
Asymp. Sig. (2-tailed)	**0.000**	0.741	0.947	0.567	**0.008**	**0.002**	**0.000**	**0.004**	0.542	0.146

Table S4. Results of the T-test showing differences (p-value: ≤ 0.05 highlighted) between the two varieties of *Beta* resulting from hierarchical clustering using IBM SPSS Software, the most significant traits (p-value: ≤ 0.01) are red colored.

Trait	Plant length	Stalk length	Lower leaf lamina length	Lower leaf lamina width	Lower leaf petiole width	Upper leaf lamina length	Upper leaf petiole length	Upper leaf petiole width	Inflorescence bract lamina length	Inflorescence bract lamina width	Inflorescence bract petiole length	Lower glomerule bract lamina length	Lower glomerule bract lamina width
Abbreviation	PL	SL	L3L	L3W	LLPW	ULLL	ULPL	ULPW	IBLL	IBLW	IBPL	LGBLL	LGBLW
Sig. (2-tailed)	0.063	0.866	**0.006**	0.110	**0.032**	**0.000**	**0.002**	**0.006**	**0.001**	**0.004**	**0.002**	**0.005**	**0.028**

Continued Table S4.

Trait	lower glomerule length	lower glomerule width	Lower glomerule bract length/Glomerule length	Upper glomerule bract lamina length	Upper glomerule bract lamina width	Upper glomerule bract petiole length	Upper glomerule length	Upper glomerule width	Upper glomerule bract length/ Glomerule length	Number of inflorescence/Branch	Number of glomerules/Inflorescence
Abbreviation	LGL	LGW	LGBL/GL	UGBLL	UGBLW	UGBPL	UGL	UGW	UGBL/GL	NInfB	NGInf
Sig. (2-tailed)	0.645	0.330	**0.008**	0.544	0.579	0.193	**0.016**	0.305	0.196	0.505	0.524
